# The Evolution of the Mental Health–Acute Coronary Syndrome Intersection: A 50-Year Bibliometric Mapping and Changepoint Analysis (1975–2025)

**DOI:** 10.3390/healthcare14081115

**Published:** 2026-04-21

**Authors:** Alexandra Herlaș-Pop, Andrei-Flavius Radu, Ada Radu, Gabriela S. Bungau, Delia Mirela Tit, Cristiana Bustea, Elena Emilia Babes

**Affiliations:** 1 Doctoral School of Biological and Biomedical Sciences, University of Oradea, 410087 Oradea, Romania; pop.alexandra@student.uoradea.ro (A.H.-P.); gbungau@uoradea.ro (G.S.B.); dtit@uoradea.ro (D.M.T.); cbustea@uoradea.ro (C.B.); eebabes@uoradea.ro (E.E.B.); 2 Department of Preclinical Disciplines, Faculty of Medicine and Pharmacy, University of Oradea, 410073 Oradea, Romania; 3 Department of Psycho-Neuroscience and Recovery, Faculty of Medicine and Pharmacy, University of Oradea, 410073 Oradea, Romania; 4 Department of Pharmacy, Faculty of Medicine and Pharmacy, University of Oradea, 410028 Oradea, Romania; 5 Department of Medical Disciplines, Faculty of Medicine and Pharmacy, University of Oradea, 410073 Oradea, Romania

**Keywords:** acute coronary syndrome, depression, anxiety, mental health, psychocardiology, bibliometric analysis, changepoint detection, PELT

## Abstract

**Background/Objectives**: The intersection of mental health and acute coronary syndromes has become an increasingly prominent area of cardiovascular and psychosomatic research, yet its temporal dynamics and intellectual structure remain incompletely characterized. **Methods**: This study analyzed 13,646 peer-reviewed documents spanning five decades, employing advanced changepoint detection (PELT) algorithms, network visualization (VOSviewer), and bibliometric performance metrics (Bibliometrix) to quantify the evolution of the mental health–ACS intersection. **Results**: Statistical analysis identified two robust inflection points at 1990 and 2005 that demarcate distinct developmental periods. The 1990 breakpoint marked an important transition, although additional metadata-completeness analysis indicated that part of the increase from 72 to 142 publications may reflect improved availability of non-title Topic-field metadata in WoSCC around 1990–1991. The 2005 breakpoint represented the most critical transition (Cohen’s d = 4.05, *p* < 0.000001), initiating exponential growth from 349 to over 600 annual publications by 2022 and coinciding with growing research attention to psychiatric comorbidity within ACS literature. Keyword co-occurrence networks revealed a shift in research focus: early publications predominantly addressed mental health as a psychological reaction to cardiac events, whereas more recent publications increasingly frame depression, anxiety, and PTSD alongside mechanistic constructs such as inflammatory pathways, autonomic dysfunction, and platelet reactivity. Although seminal intervention trials (i.e., ENRICHD, SADHART) established pharmacological safety and symptom improvement, keyword analyses indicate that following these trials, research attention increasingly shifted toward precision psychiatry concepts and mechanistic pathway elucidation. **Conclusions:** These findings provide a quantitative map of how publication activity at the mental health–ACS intersection has evolved, offering a structured basis for identifying under-researched areas and informing future research agendas.

## 1. Introduction

Cardiovascular disorders remain the predominant contributors to death worldwide and constitute a major source of long-term functional impairment across populations. Over the past three decades, most regions have experienced a steady escalation in cardiovascular disease prevalence and impact, largely reflecting demographic expansion, progressive population aging, and persistent exposure to deleterious behavioral and metabolic risk factors. Contemporary patterns of cardiovascular burden arise from multifactorial interactions that include modifiable lifestyle determinants, sociodemographic transitions, and heterogeneity in healthcare availability and quality [1,2].

Within this broad cardiovascular landscape, acute coronary syndromes (ACSs) represent one of the most clinically consequential and resource-intensive manifestations. ACSs continue to represent a major source of mortality and functional impairment. Recent guideline revisions and high-quality studies, including the 2023 European Society of Cardiology ACS document and updated European Society of Cardiology/European Atherosclerosis Society dyslipidaemia recommendations, have further refined diagnostic pathways, revascularization decision-making, and pharmacological management across the ACS spectrum [3].

Globally, ACS mortality remains consistently higher in men than in women. Currently, the greatest age-standardized mortality rates occur in lower-income regions, whereas two decades earlier the highest burden was concentrated in high-income areas. Mortality has declined markedly in wealthier countries but shows limited improvement in many parts of Asia and Latin America, with modest increases reported in some African settings. These divergent trajectories underscore the need for improved epidemiological surveillance to identify priority regions for targeted prevention [4].

Evidence synthesized from large-scale analyses indicates that multiple categories of mental illness, including depressive and anxiety disorders, post-traumatic stress disorder (PTSD), and disturbances of sleep, are linked to a heightened likelihood of developing ACS. Among these conditions, PTSD and sleep-related disorders show the most consistent and robust associations, underscoring the relevance of sleep integrity in cardiovascular vulnerability. Further investigations designed to overcome current methodological constraints are warranted to clarify the complexity of interactions between psychological pathology and ACS [5].

Following an acute coronary event, substantial changes occur in both physical performance and overall quality of life. Levels of functional fitness strongly influence subsequent depressive and anxiety symptoms, emotional burden, social participation, and posttraumatic adjustment. These patterns underscore a reciprocal relationship in which somatic impairment and psychological status continuously interact after ACS [6].

Elevated depressive and anxiety symptoms after ACS are also linked to slower clinical recovery, increased mortality and rehospitalization, poorer quality of life, and accelerated coronary disease progression. Pre-existing depression confers higher one-year post-ACS mortality compared with absence of prior depressive illness. Targeting psychological distress within secondary prevention frameworks may therefore enhance long-term prognosis and patient-centered outcomes, although the most effective therapeutic approach remains uncertain [7].

Greater clinical burden and elevated psychological stress were linked to poorer health-related quality of life among individuals with ACS, with these associations operating through patients’ perceptions of their illness [8]. Clinical judgment alone may be insufficient to reliably identify clinically relevant depressive burden in ACS. The integration of standardized instruments such as PHQ9 and formal psychological assessment can improve diagnostic precision. Such multidimensional evaluation is essential for delivering comprehensive care and optimizing outcomes [9].

In the cardiovascular literature, depression has been operationalized using multiple approaches rather than a single uniform definition. Thombs et al. reviewed depression screening instruments in cardiovascular care and found that diagnostic accuracy varied across instruments and cutoff thresholds. No studies assessing whether screening for depression improved depressive symptoms or cardiac outcomes in patients with cardiovascular disease have been found [10]. Gan et al. included prospective cohort studies in which depression was defined by self-reported scales, clinician/physician diagnosis, or structured clinical diagnostic interview, and they noted heterogeneity across studies that might reflect differences in participant characteristics, study design, and depression diagnostic criteria. Because most included studies used self-reported symptom scales, Gan et al. stated that their findings are more generalizable to populations with depressive symptoms than to populations defined only by formal interview-based diagnostic criteria [11].

Given this underrepresented association as a critical gap, bibliometric analyses become increasingly important for characterizing the expanding mental health–ACS interface, as they quantify knowledge growth, thematic concentration, and emerging research directions. In the psychocardiology space, a prior bibliometric complex work has largely mapped broader coronary heart disease research linking depression or anxiety, rather than isolating ACS phenotypes across the full care continuum [12]. A separate bibliometric analysis has also examined psychocardiology as a wider field, confirming rapid growth and thematic diversification, but without focusing specifically on ACS-defined trajectories [13]. However, the ACS-specific bibliometric mapping of psychiatric comorbidity remains limited, despite strong clinical rationale for continuous research to improve risk stratification, treatment response, and outcomes.

As ACS management increasingly shifts toward individualized, mechanism-guided strategies, mental health comorbidities have emerged as critical modifiers of risk and recovery, yet their scientific integration remains heterogeneous. Mapping how this evidence has evolved over time is essential to identify conceptual shifts while the ongoing necessity of investigating these associations to improve clinical outcomes and patient management must be anchored in structured scientific literature, utilizing performance-based bibliometric indicators as an objective foundation [3].

This study aims to generate a reproducible, data-driven overview of advances in psychocardiology by characterizing how mental health comorbidity has been addressed within ACS research over five decades. A central objective is the application of PELT changepoint detection to define developmental phases from publication time series, replacing arbitrary temporal partitioning with statistically derived segmentation. To our knowledge, this represents the first ACS–mental health bibliometric analysis to implement PELT for breakpoint identification, enhancing temporal resolution and reproducibility. By integrating changepoint modeling with keyword co-occurrence networks and performance indicators, this work seeks to contextualize evolving research priorities and support ongoing surveillance of the ACS–mental health literature.

## 2. Materials and Methods

To investigate the interdisciplinary connection between psychological health and cardiovascular outcomes, this study analyzed the bibliometric data regarding the impact of mental disorders on Acute Coronary Syndromes (ACSs). The data acquisition was restricted to the Web of Science Core Collection (WoSCC). While multi-database aggregation (e.g., combining Scopus and WoS) is common in general reviews, this study avoided such merging to preserve the fidelity of the temporal metadata. Uniform indexing protocols are a prerequisite for the advanced changepoint detection algorithms employed in this research, as merging disparate databases often introduces time-series artifacts and inconsistent metadata structures that obscure genuine statistical breakpoints [14,15].

The search was conducted across all available sub-indexes of the WoSCC, in accordance with the transparency requirements outlined by Liu (2019) [16]: Science Citation Index Expanded (SCI-EXPANDED, 1975–present), Social Sciences Citation Index (SSCI, 1975–present), Arts & Humanities Citation Index (AHCI, 1975–present), Conference Proceedings Citation Index—Science (CPCI-S, 1990–present), Conference Proceedings Citation Index—Social Science & Humanities (CPCI-SSH, 1990–present), Book Citation Index—Science (BKCI-S, 2010–present), Book Citation Index—Social Sciences & Humanities (BKCI-SSH, 2010–present), Emerging Sources Citation Index (ESCI, 2006–present), Current Chemical Reactions (CCR-EXPANDED, 2010–present), and Index Chemicus (IC, 2010–present). Although some of these sub-indexes, such as AHCI, CCR-EXPANDED, and IC, are only marginally relevant to the psychocardiology field, they were retained to reflect the full WoS search framework and to ensure transparent reporting of the data retrieval process. To maintain the scientific relevance of the final dataset, the results were subsequently limited to peer-reviewed Articles and Review Articles, which reduced the inclusion of records from less relevant or non-clinical indexes. It should also be noted that sub-indexes introduced after 1975, especially ESCI (from 2006 onward) and BKCI (from 2010 onward), may have contributed to the apparent increase in publication output in more recent years. However, because the search strategy was narrowly focused on acute coronary syndrome and mental health-related terms, the influence of these later-added indexes on the overall dataset is likely to be small.

Both Web of Science and Scopus are widely recognized as the primary databases for large-scale bibliometric research [17,18]. WoSCC was selected as the data source for this study due to its established use in prior bibliometric studies within the psychocardiology domain [12,13], its compatibility with the analytical tools employed (i.e., Bibliometrix and VOSviewer), and to ensure the metadata uniformity required by the PELT changepoint detection algorithm.

The identification of relevant documents was facilitated by a specialized search algorithm designed to capture the intersection of psychopathology and cardiovascular medicine. To minimize false positives and ensure the retrieval of high-fidelity data, the query utilized Boolean operators (OR within domains, AND between domains) alongside exact phrase matching (enclosing compound terms in quotation marks) and wildcard operators (*) to capture morphological variations (e.g., “disorder” vs. “disorders”). The final search algorithm deployed for data extraction was structured as follows:

- TS = (“mental health” OR “mental disorder*” OR “mental illness*” OR “psychiatric disorder*” OR “psychological disorder*” OR depression OR depressive OR anxiety OR “anxiety disorder*” OR “post-traumatic stress” OR PTSD OR “psychological stress” OR “psychological distress” OR “bipolar disorder” OR schizophreni* OR psychosis OR “mood disorder*” OR “affective disorder*” OR “panic disorder” OR phobia* OR “obsessive-compulsive” OR OCD)

AND

- TS = (“acute coronary syndrome*” OR ACS OR “unstable angina” OR NSTEMI OR “non-ST-segment elevation myocardial infarction” OR “non-ST elevation myocardial infarction” OR STEMI OR “ST-segment elevation myocardial infarction” OR “ST elevation myocardial infarction” OR “myocardial infarction” OR “heart attack” OR “acute myocardial infarction” OR AMI).

This comprehensive string queried the “Topic” (TS) field, encompassing titles, abstracts, author keywords, and keyword plus. The strategy was refined to prioritize precision; for instance, exact phrase matching for terms like “post-traumatic stress” was necessary to eliminate irrelevant records that might contain these words in disjointed contexts.

The mental health component of the search strategy was intentionally designed to capture a broad conceptual field rather than only formal DSM-5 diagnostic entities. Accordingly, the search terms were interpreted in three categories: (1) diagnostic constructs, including terms corresponding to established psychiatric disorders such as anxiety disorders, post-traumatic stress disorder, bipolar disorder, schizophrenia-spectrum disorders, panic disorder, phobic disorders, and obsessive–compulsive disorder; (2) symptom-level constructs, including depression, depressive symptoms, anxiety, and related dimensional symptom expressions that are often operationalized in cardiovascular research through self-report scales rather than structured diagnostic interviews; and (3) broad non-diagnostic psychosocial constructs, including mental health, psychological stress, and psychological distress. These categories were considered conceptually related but not diagnostically equivalent, and the findings were interpreted accordingly at the bibliometric level.

Because the historical completeness of Topic-field metadata in WoSCC is uneven for older records, an additional sensitivity analysis was performed to evaluate the potential effect of metadata availability on temporal trends. This analysis was motivated by prior methodological work showing that historical bibliometric series based on WoSCC Topic searches may be affected by incomplete indexing of abstracts, author keywords, and Keywords Plus, particularly around the 1990–1991 transition [19]. Using the retrieved dataset, we quantified yearly metadata completeness for the publication year, title, abstract, author keywords, and Keywords Plus fields and compared records published before 1991 with those published from 1991 onward. For each period, we calculated the proportion of records containing abstracts, author keywords, and Keywords Plus, as well as the proportion containing all three non-title Topic-field components. This procedure was intended to assess whether the apparent increase in publication output around 1990–1991 might partly reflect improved metadata coverage in WoSCC rather than only genuine growth in the field.

The sensitivity analysis was implemented using a custom Python 3.12.3 script (code available at https://github.com/AndreiUO/wos-metadata-sensitivity-analysis (accessed on 3 March 2026)) applied to all Web of Science export files. The script imported the records from a user-specified directory, prioritizing tab-delimited WoS exports and preserving the original field structure, while identifying the key metadata columns for publication year, title, abstract, author keywords, Keywords Plus, DOI, and accession number. After merging the records into a single dataset, duplicate entries were removed hierarchically using accession number (UT) as the primary identifier, DOI as the secondary identifier, and normalized title plus publication year as a fallback rule. Binary completeness indicators were then generated for abstracts, author keywords, and Keywords Plus, and these were used to calculate yearly metadata coverage and period-based comparisons, with particular emphasis on pre-1991 versus 1991-and-later records. The script also exported the cleaned dataset, summary tables, statistical comparisons, parsing logs, and graphical outputs used to evaluate metadata discontinuities around the 1990–1991 breakpoint.

**Figure 1:**
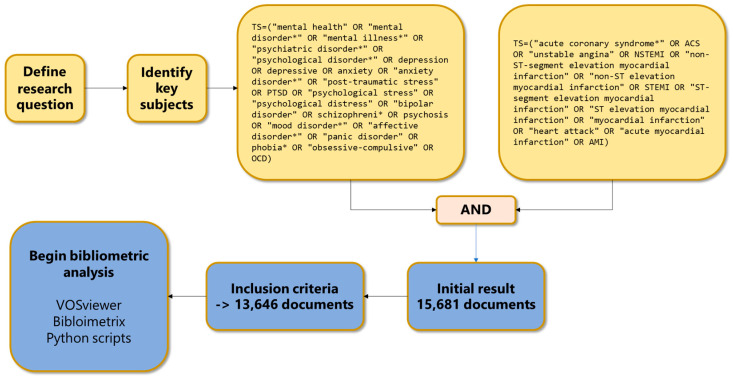
A multi-stage query refining process illustrating the systematic literature search and filtering methodology for mental health conditions and acute coronary syndrome bibliometric analysis (Web of Science Core Collection, 1975–2025).

The temporal scope of the analysis was strictly delimited to documents published up to the year 2025. This distinct temporal cutoff was imposed to ensure the reproducibility of the study; unlike open-ended searches that fluctuate with daily database updates, a fixed end-year creates a static, verifiable dataset essential for the consistent application of changepoint detection algorithm. The systematic search and selection process is illustrated in Figure 1, demonstrating the multi-stage query refinement approach that yielded the final dataset of 13,646 documents (exported 24 January 2026).

The execution of this optimized search protocol yielded a final analytical corpus of 13,646 documents. While preliminary exploratory searches utilizing unconstrained wildcard operators suggested a significantly larger volume of literature, a qualitative inspection revealed a high prevalence of “noise”, specifically, records where terms like “stress” or “depression” appeared in mechanical or economic contexts rather than clinical ones. Consequently, the strict application of exact phrase matching was essential to sanitize the dataset. Following the exclusion of non-English records and the restriction of document types to peer-reviewed “Articles” and “Review Articles,” the resulting collection represents a high-fidelity sample of the intersection between psychopathology and acute coronary syndromes. This rigorous selection process ensures that the subsequent computational analyses (e.g., PELT) operate on relevant data, thereby preventing the distortion of statistical changepoints by irrelevant false positives.

The architectural framework of this bibliometric analysis relied on a specific suite of computational tools selected to ensure both the reproducibility of the statistical metrics and the clarity of the network visualizations. The construction of bibliometric maps was executed using VOSviewer (version 1.6.20), which facilitated the visualization of the intellectual structure connecting the psychiatric and cardiovascular domains through co-authorship and keyword co-occurrence networks. For the quantitative assessment of scientific performance, the study employed the Bibliometrix library (version 4.3.0) within the RStudio environment (version 4.4.2), this was accessed primarily through the Biblioshiny web interface to compute performance indicators such as the “Mean Total Citations per Year” and to track publication velocity. Microsoft Excel served as the intermediary tool for preliminary data sanitization and the generation of linear temporal graphs. Crucially, to overcome the limitations of standard software in handling inconsistent medical terminology, custom scripts were developed in Python (version 3.12.3) [20,21,22].

Publication patterns of top-contributing countries were visualized using Python 3.12.3, leveraging the Pandas (v2.3.1) [23], Matplotlib (v3.10.3) [24], Seaborn (v0.13.2) [25], and NumPy (v2.3.1) libraries [26]. Data were exported from the Bibliometrix interface, after which total publication counts were stratified by year and country. Heat maps were generated to illustrate research activity intensity, incorporating grid lines and five-year *x*-axis intervals to enhance legibility. This visualization strategy facilitated the identification of longitudinal trends and the evaluation of national research output variations over the 51-year study period.

To quantifiably map the trajectory of the research field, this study analyzed two primary vectors: publication velocity and normalized citation impact. The annual volume of scientific production was extracted via the Bibliometrix library, with subsequent linear temporal trend visualizations generated using Microsoft Excel. A critical challenge in bibliometrics is comparing the impact of older foundational papers against recent publications that have had less time to accrue citations. To address this, the study employed the Mean Total Citations per Article (MeanTCperArt) metric alongside citable years analysis. This indicator measures the average number of citations received per publication within each annual cohort, enabling assessment of research influence while acknowledging that citation accumulation is inherently time-dependent. These values were computed automatically through the Biblioshiny web interface, ensuring mathematical consistency across the dataset. By presenting MeanTCperArt in conjunction with citable years—representing the time elapsed since publication, the analysis allows for contextualized interpretation of citation patterns, recognizing that recent publications naturally exhibit lower citation counts due to insufficient accumulation time rather than diminished scientific relevance. To contextualize the growth of mental health-related ACS research within the broader cardiovascular literature, a proportional analysis was conducted comparing publication volumes against total ACS research output. Total ACS research was operationally defined using a modified search query that retained only the cardiovascular component of the original algorithm, excluding mental health terminology. The annual share of mental health-focused ACS research was calculated by dividing the number of publications addressing the mental health and ACS intersection by the total ACS publications for each year, expressed as a percentage. This methodological approach enables differentiation between absolute growth attributable to general expansion in ACS research and genuine increases in relative research attention toward mental health dimensions, thereby providing insight into evolving research priorities within the cardiovascular field.

To establish an objective chronological framework for the analysis, rather than relying on arbitrary decadal segmentation (e.g., 2000—2010), this study utilized the Pruned Exact Linear Time (PELT) algorithm to detect structural breaks in the bibliographic time series. The algorithm was implemented using the Python ruptures library (version 1.1.0) with a Radial Basis Function (RBF) cost model. The RBF-based kernel cost was selected because kernel change-point detection provides a non-parametric framework that does not require specifying a parametric distribution family in advance. In the Gaussian/RBF case, changes in the underlying distribution can be represented as mean shifts in a transformed feature space, making the method suitable for detecting a broad class of regime changes [27].

In PELT, the penalty parameter controls the trade-off between model fit and model complexity: lower penalties favor more breakpoints, while higher penalties favor fewer. To ensure the robustness of the detected transitions, a sensitivity analysis was conducted by varying the penalty parameter, which regulates the algorithm’s responsiveness to fluctuations, across a range of values from 1 to 5. This range was chosen to span from a liberal setting (penalty = 1), which risks over-segmentation, to a conservative setting (penalty = 5), which retains only the most prominent structural shifts. At penalty values ≥ 3 the algorithm converged on two breakpoints (1990 and 2005); these same breakpoints were also detected at penalties 1 and 2, alongside additional, less stable changepoints. A breakpoint was only accepted as a valid evolutionary milestone if it was consistently identified in at least three of the five sensitivity simulations. The additional changepoints at 2000 and 2010 were not retained because they were penalty-sensitive and therefore did not satisfy the predefined robustness criterion.

Following this detection phase, the statistical significance of the identified periods was verified using the Mann–Whitney U test (via scipy 1.9.0) to confirm that the publication volumes between consecutive phases represented distinct, statistically differentiable populations. The Mann–Whitney U test was selected over parametric alternatives because it does not assume normality within segments, which is appropriate given the small segment sizes (n = 15–20 annual observations per phase) and the positively skewed distribution of publication counts. Effect sizes were quantified using Cohen’s d, calculated as the difference in segment means divided by the pooled standard deviation, to characterize the practical magnitude of each transition. It should be noted that the Mann–Whitney U test assumes independence of observations; because annual publication counts within a segment may exhibit temporal autocorrelation, the reported *p*-values should be interpreted as approximate indicators of separation strength rather than exact Type I error probabilities. The very large effect sizes (Cohen’s d > 3.9 for both transitions) and complete rank separation between adjacent segments (rank-biserial r = 1.0) provide convergent evidence that the detected breakpoints reflect substantive discontinuities in publication activity.

The country collaboration network included only nations with at least 20 publications to ensure reliable results. In the visualization, circle size represents total publication output, while line thickness indicates collaboration strength between countries. Country name variations were manually standardized (e.g., merging “ Turkiye” and “Turkey”) to ensure data accuracy. Terms are clustered when they share identical normalized forms (e.g., ‘cardiovascular-disease’ and ‘cardiovascular disease’) or exceed strict dual thresholds for both hybrid and string similarity. The final parameter configuration, comprising a hybrid similarity threshold of 0.90, a string similarity threshold of 0.90, and a semantic weight of 0.40, was established through iterative empirical refinement against domain-specific requirements. Validation was conducted by three independent human reviewers who assessed a reproducible sample of 300 candidate term pairs generated from the tuned thesaurus. Each pair was classified as ‘merge accepted’ or ‘merge rejected’ according to whether replacement preserved the same biomedical concept for thesaurus normalization. Inter-rater agreement was high (Fleiss’ kappa = 0.9041). Final adjudication was performed by majority vote, yielding 271 accepted merges and 29 rejected merges (90.33%; 95% CI, 87.14–93.53); 292 pairs showed unanimous agreement and 8 were resolved by a 2:1 majority, with no manual overrides. Accepted merges included orthographic, formatting, or equivalent biomedical variants such as ‘life-style’ versus ‘lifestyle’, ‘marital-status’ versus ‘marital status’, and ‘cardiovascular-disease’ versus ‘cardiovascular disease (CVD)’. Rejected merges included concept-changing or artifact-driven pairs such as ‘coronary thrombus’ versus ‘coronary thrombosis’, ‘psychiatric’ versus ‘psychiatry’, and ‘high-resolution electrocardiogram’ versus ‘high resolution electrocardiography’. Initial configurations employing a lower similarity threshold (0.75) and higher semantic weight (0.60) resulted in systematic false positives, erroneously clustering semantically related but distinct medical entities, such as conflating ‘depression’ with ‘depressive symptoms’. Reducing the semantic weight to 0.40, while correspondingly increasing the emphasis on string similarity, shifted the model toward conservative lexical normalization. This ensured that clustering decisions prioritized orthographic correspondence over broader semantic proximity and reduced false-positive conflation of related but non-equivalent clinical constructs, such as ‘acute coronary syndrome’ and ‘acute myocardial infarction’.

This study presents a hybrid approach for automated thesaurus generation designed to normalize keyword variants in bibliometric analyses of biomedical literature (code at https://github.com/AndreiUO/thesaurus-mental-health-acs (accessed on 4 March 2026)). The methodology combines transformer-based semantic embeddings with traditional string similarity metrics to identify term variants while preserving conceptually distinct entities. Specifically, we employ SapBERT (Self-Alignment Pretraining for BERT), a model fine-tuned on the Unified Medical Language System (UMLS), to produce semantically aligned representations for biomedical synonyms, paired with Jaro-Winkler string similarity to capture surface-level variations such as hyphenation [28,29].

Terms are clustered when they share identical normalized forms (e.g., “cardiovascular-disease” and “cardiovascular disease”) or exceed strict dual thresholds for both hybrid and string similarity. The final parameter configuration, similarity threshold of 0.90, string similarity threshold of 0.90, and semantic weight of 0.40 emerged through iterative empirical refinement against domain-specific requirements.

**Figure 2:**
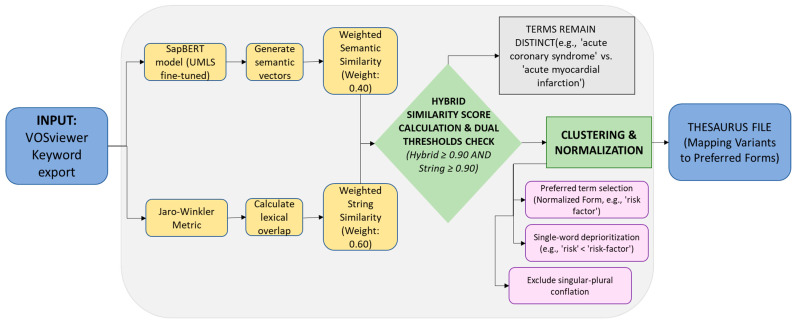
Architecture of the dual-path similarity assessment combining semantic (SapBERT) and lexical (Jaro-Winkler) metrics.

Further refinements were made to address specific linguistic challenges. The preferred term selection algorithm was modified to prioritize normalized forms (replacing hyphens with spaces) over simple high-frequency variants, yielding more readable canonical terms for visualization tools like VOSviewer. Additionally, generic single-word terms (e.g., “risk”) were deprioritized when multi-word variants (e.g., “risk-factor”) existed in the cluster. Finally, automatic singular-plural conflation was deliberately excluded; consultation with domain experts revealed that grammatical number often carries semantic significance in medical literature (e.g., “risk factor” as a specific element vs. “risk factors” as a broader study), warranting separate treatment in bibliometric analyses. Figure 2 visualizes the complete processing pipeline, detailing the integration of SapBERT embeddings and Jaro-Winkler similarity with the applied filtration thresholds.

## 3. Results

### 3.1. Temporal Publication Trends and Citation Dynamics

**Figure 3:**
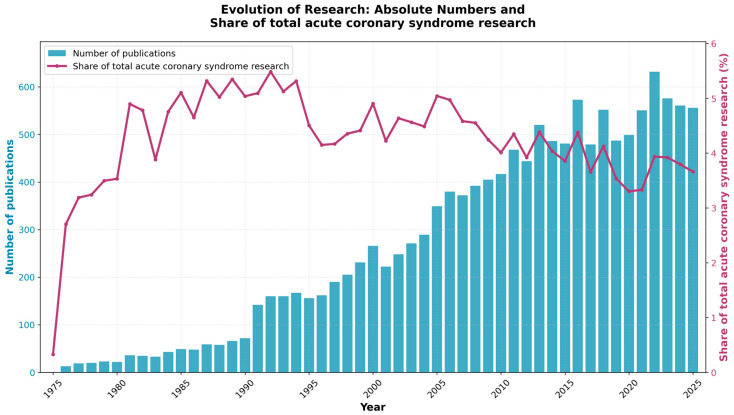
Publication trends in mental health–ACS research: absolute output and proportional share of total ACS literature (1975–2025).

The analysis covers 13,646 publications from 1975 to 2025, fifty years of research that reflects how scientific interest in the link between mental health and acute coronary syndrome has grown substantially over time. The temporal distribution (Figure 3) reveals remarkable growth in research output, with annual publications increasing from a single article in 1975 to 632 articles at the peak year of 2022, representing a significant increase.

A notable inflection point occurred between 1990 and 1991, when annual output increased from 72 to 142 publications. To assess whether this rise reflected only substantive growth or was also influenced by database characteristics, we performed an additional metadata-completeness sensitivity analysis. Among pre-1991 records (n = 597), abstracts were available for 570 records (95.48%), whereas author keywords and Keywords Plus were present in only 7 (1.17%) and 12 (2.01%) records, respectively. In contrast, among records from 1991 onward (n = 13,049), abstract coverage remained high (98.28%), while author keywords and Keywords Plus increased sharply to 75.35% and 96.54%, respectively. The most pronounced discontinuities occurred between 1990 and 1991 for author keywords (9.72% vs. 42.96%) and Keywords Plus (12.50% vs. 91.55%), indicating that part of the apparent post-1990 increase in Topic-retrieved literature may reflect improved metadata availability in WoSCC rather than only true growth in the field.

The proportional analysis, comparing mental health-focused ACS research against total ACS literature, reveals that this research domain has significantly increased its relative share from 0.3% in 1975 to 3.7% in 2025, indicating increased publication attention beyond the general expansion of ACS research. Notably, the relative research attention peaked at 5.5% in 1992, reflecting a period when foundational studies establishing the mental health–cardiac connection commanded substantial attention within a comparatively smaller ACS literature base. The subsequent decline in proportional share, despite continued absolute growth, reflects the exponential expansion of total ACS research during the interventional cardiology and biomarker eras. The most recent five-year average has stabilized at approximately 3.7%, suggesting relatively stable representation of this topic within the indexed ACS literature. The year 2022 marked the highest absolute output (632 publications), though 2024–2025 data suggest a modest plateau around 560 annual publications.

**Figure 4:**
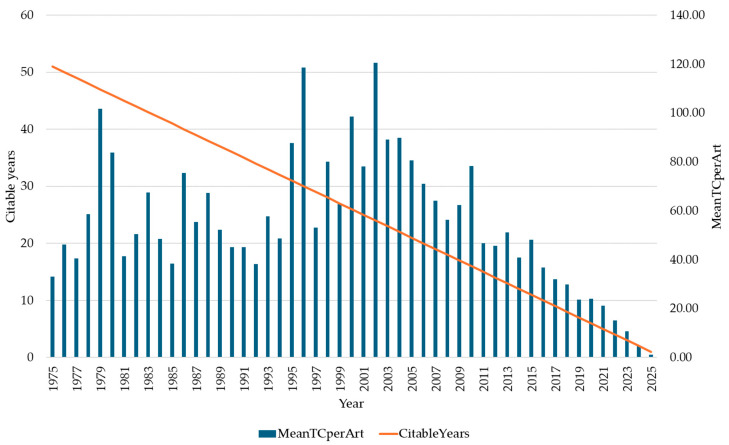
Mean citations per article and citable years in research examining the mental health–acute coronary syndrome intersection (1975–2025).

A five-decade analysis of citation patterns (Figure 4) illustrates the scientific maturation and growing influence of research at the intersection of mental health disorders and ACS. The MeanTCperArt reveals distinct phases of impact, with the field reaching its apex in 2002 (120.46 citations) and 1996 (118.56 citations). Secondary citation peaks occurred in 1979 (101.65 citations), 2000 (98.55 citations), and during 2003–2004 (89.04 and 89.84 citations, respectively), indicating sustained high-impact research throughout this period.

The early decades of the dataset (1975–1994) exhibited considerable variability in citation impact, ranging from 33.00 citations per article in 1975 to 101.65 in 1979, reflecting the exploratory nature of research during this period. In contrast, the 1995–2010 interval demonstrated consistently elevated citation rates, with multiple years exceeding 60 citations per article, suggesting a period of sustained scientific influence and growing recognition of the mental health–acute coronary syndrome intersection within the broader medical literature.

Citation rates have dropped steadily since 2011, falling from 46.72 to just 1.15 by 2025. This reflects the natural lag in citation accumulation rather than any decline in research quality. Recent papers simply have not had enough time to gather citations yet. The sustained citation activity during this period is consistent with a consolidation of scholarly interest in the mental health–ACS connection, suggesting that the topic has become an established research area within the broader cardiovascular literature.

### 3.2. Geographic Distribution and National Research Contributions

Table 1 illustrates the geographic dominance of the United States, which accounts for 5233 documents, over four times the output of the second-ranked nation. The U.S. also leads in total citations (345,358) and collaboration strength (TLS of 2480), with a mean impact of 66.00 citations per document. However, the Netherlands and Canada exhibit superior citation efficiency, recording averages of 67.54 and 67.08, respectively. The United Kingdom presents a well-rounded profile of high volume (1263) and impact (64.87), alongside a strong collaborative network (TLS of 1404).

European nations provide steady contributions, led by Germany (741 documents; 61.08 citations/document), Sweden (609; 48.60), and Italy (595; 49.83). In contrast, China’s rapid growth in volume (917 documents; 4th overall) is not yet matched by influence, evidenced by a lower citation average (23.34) and moderate collaboration (TLS of 532). Japan follows a similar trajectory, with 422 documents, an average of 29.73 citations, and the cohort’s lowest collaboration score (TLS of 194).

**Figure 5:**
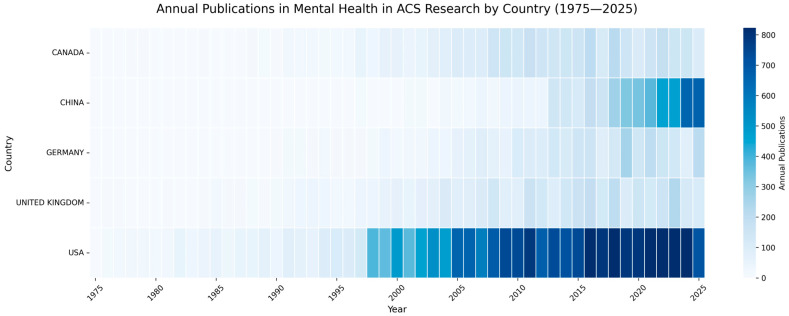
Heat map of global research output on mental health and acute coronary syndrome from 1975 to 2025. Each cell displays the number of publications per country for a given year, with darker blue shades indicating higher output. Years appear on the horizontal axis at five-year intervals, while countries are listed vertically. The visualization was created in Python using the Seaborn and Matplotlib libraries. Note: The cumulative counts here represent running totals of country-attributed documents over time.

Figure 5 illustrates the longitudinal trends in research output from 1975 to 2025, highlighting a transition from Western-centric production to a multipolar research environment. The foundational period (1975–2000) was defined by U.S. leadership (2404 cumulative publications), with secondary contributions from the United Kingdom (463), Canada (365), and Germany (171). Because multi-national collaborations are counted once for each contributing country, the figure differs from the unique document counts shown in Table 1.

In the contemporary period, China demonstrates the most significant rate of change. Starting from a baseline of near-zero output in 2000 (9 cumulative publications), China’s trajectory shifted to rapid acceleration, particularly between 2015 and 2025, where cumulative publications rose more than six-fold (710 to 4603). By 2025, China effectively surpassed the cumulative totals of the UK (3525), Canada (3630), and Germany (2861).

Despite this competitive surge, the United States retained its dominant position, increasing its cumulative output to 20,325 by 2025. These data suggest that while the field is witnessing the rapid entry of Asian research hubs, the sheer volume of U.S. scholarship continues to outpace all other contributors by a significant margin.

### 3.3. Publication Venues and Institutional Contribution

Circulation demonstrates the strongest bibliometric performance within the mental health–acute coronary syndrome domain, leading in both total impact and visibility (210 publications; 37,818 citations, Table 2). Its dominance is quantified by a field-leading h-index of 101 and g-index of 194. However, regarding citation accumulation rates, Psychosomatic Medicine surpasses all peers with an m-index of 2.415 across 353 articles, underscoring its role as the central hub for interdisciplinary research.

The American Journal of Cardiology and JACC represent key pillars of research volume and impact, contributing 358 and 195 publications, and achieving h-indices of 71 and 74, respectively. Historical continuity is provided by the Journal of Psychosomatic Research and American Heart Journal, which have maintained consistent output since 1976.

A distinct ‘high-efficiency’ pattern is observed in generalist journals. JAMA and Archives of Internal Medicine achieved disproportionately high citation counts (16,734 and 12,076) relative to their limited publication volumes (45 and 71, respectively). This suggests that while specialized journals drive the bulk of the conversation, generalist platforms are critical for the broad dissemination of landmark studies.

**Figure 6:**
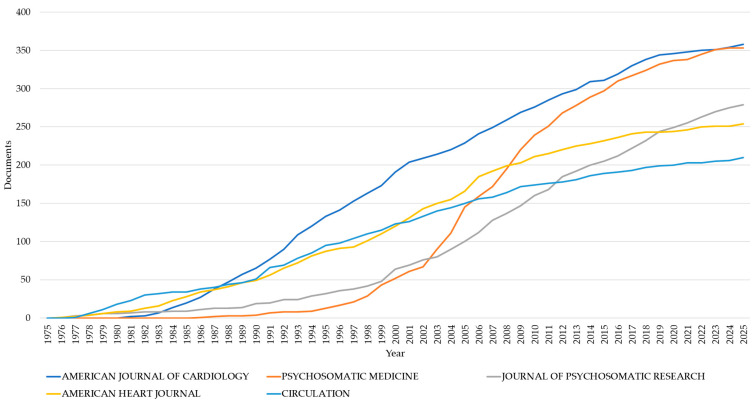
Comparative publication dynamics among leading journals in mental health–cardiovascular research (1975–2025).

Figure 6 visualizes the maturation of the mental health–acute coronary syndrome research landscape. Long-standing engagement is evident in the Journal of Psychosomatic Research and American Heart Journal (active since 1976; 279 and 254 cumulative publications by 2025, respectively). The cardiology sector also maintained robust output, with Circulation reaching 210 publications by 2025 and the American Journal of Cardiology achieving the highest total volume (358) after growing steadily from its 2000 baseline of 191.

However, Psychosomatic Medicine (active since 1986) demonstrates the most dynamic growth. Its cumulative output increased nearly seven-fold between 2000 and 2025 (from 52 to 353 publications). Notably, a surge between 2003 and 2016 saw publications triple from 90 to 310. These trends suggest a bifurcation in the literature: established cardiology journals provide a steady stream of research, while specialized psychosomatic outlets have rapidly expanded to meet the increasing recognition of the mental health–ACS nexus.

**Figure 7:**
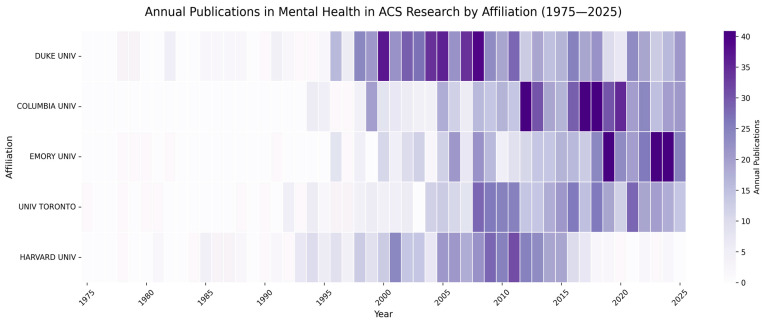
Academic institution publication dynamics in mental health–ACS research (1975–2025).

Heatmap analysis (Figure 7) reveals significant heterogeneity in institutional momentum. Duke University leads the cohort with 686 cumulative publications by 2025, driven by a major acceleration phase beginning in 1996. The University of Toronto, representing the longest-standing contributor (entry: 1975), demonstrated consistent linear growth, rising from 40 publications in 2000 to 463 by 2025.

The highest rates of recent growth were observed at Columbia University and Emory University. Columbia’s output increased nearly four-fold between 2010 and 2025 (149 to 560 publications), with the output more than doubling between 2015 and 2025. Emory similarly expanded rapidly from 302 cumulative publications in 2019 to 486 by 2025. In contrast, Harvard University showed signs of a production plateau; after reaching 412 publications in 2016, the institution added only minimal output through 2025 (totaling 429). These data collectively indicate a restructuring of the research hierarchy, characterized by the stabilization of historical centers and the rapid ascent of contemporary hubs.

Table 3 identifies the ten most cited documents shaping the mental health–acute coronary syndrome nexus. The list is dominated by the meta-analysis of Holt-Lunstad et al. (2010), which achieved exceptional contemporary impact (Normalized Citation Score: 63; 4926 total citations) by equating social relationship quality with established mortality risks. This psychosocial perspective is reinforced by landmark works on social conditions (Link & Phelan, 1995; 4272 citations), loneliness pathways (Hawkley & Cacioppo, 2010; 2692 citations), and stigma (Corrigan, 2004; 2291 citations).

Parallel to these social frameworks, seminal biological investigations provided mechanistic validity. Lesch et al. (1996) established the genetic basis of anxiety traits via the serotonin transporter gene (4114 citations), while Raison et al. (2006) and Howren et al. (2009) elucidated the inflammatory pathways (CRP, IL-6) connecting depression to somatic disease. The corpus is rounded out by key studies on nutritional pathogenesis (Simopoulos, 2002) and global risk factors (O’Donnell et al., 2010). The diversity of publication venues, ranging from The Lancet and Science to Psychosomatic Medicine, highlight the field’s successful integration of diverse distinct scientific epistemologies.

### 3.4. Research Collaboration and Conceptual Structure Analysis

**Figure 8:**
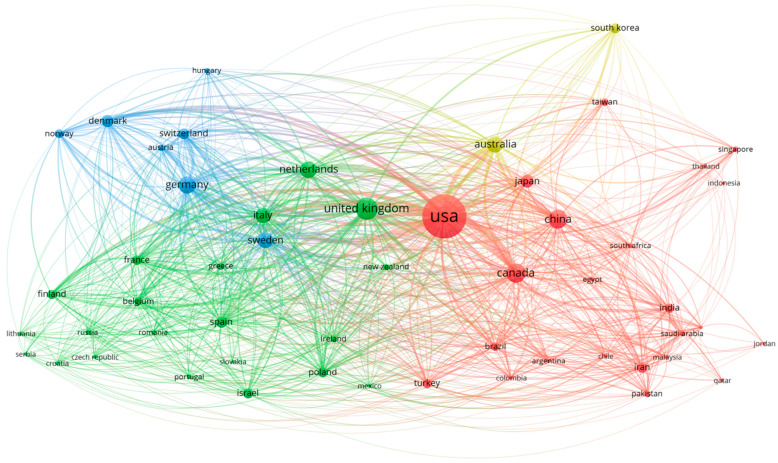
Visualization of the global collaborative network in mental health and ACS research.

The visualization of global collaboration (Figure 8) reveals a highly structured ecosystem defined by US centrality and regional European integration. The United States (5233 documents; TLS: 2480) dominates the network topography, serving as the primary hub for Cluster 1, a diverse transcontinental alliance. This cluster includes Canada, notable for its high average impact (67.08 citations/document), and emerging contributors such as China (917 documents), India, and Brazil.

European research is organized into two distinct clusters. The first, led by the United Kingdom (Cluster 2), includes Italy, France, and the high-impact research output of the Netherlands (67.54 citations/document). The second (Cluster 3) represents a tightly integrated Germanic-Nordic block led by Germany (TLS: 839), featuring strong intra-regional connectivity with Sweden, Denmark, and Switzerland.

A distinct Asia-Pacific cluster (Cluster 4), anchored by Australia (TLS: 747) and South Korea, operates at the network’s periphery. Despite its smaller size, this cluster functions as a critical bridge between established Western networks and the expanding Asian research sphere, illustrating a matured field where traditional powerhouses integrate increasingly influential global partners.

**Figure 9:**
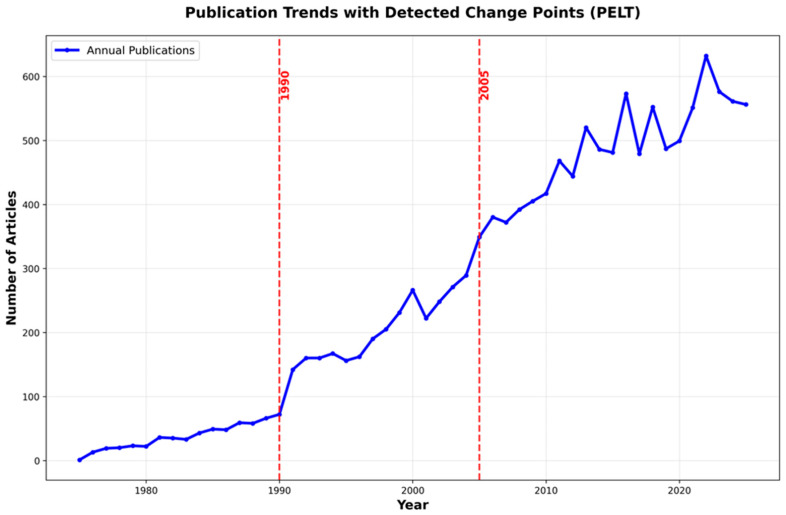
Temporal segmentation and thematic evolution of mental health in ACS research. Pruned Exact Linear Time (PELT) change point analysis of the 1975–2025 time series identifying robust breakpoints.

To avoid arbitrary temporal partitioning, a Pruned Exact Linear Time (PELT) change point detection algorithm was applied to the 1975–2025 publication time series (Figure 9). This method identified structural shifts in research intensity concerning the intersection of mental health and ACS. Sensitivity modeling across five penalty values (1–5) yielded consistent results: at penalties ≥ 3 the algorithm detected exactly two breakpoints (1990 and 2005), while at lower penalties (1–2) these same breakpoints were retained alongside additional, less stable changepoints (2000 and 2010 at penalty = 1; 2010 at penalty = 2). The two primary breakpoints were thus detected in 100% of iterations (5/5), and were the only breakpoints present across all penalty settings. By contrast, candidate breakpoints at 2000 and 2010 appeared only in lower-penalty runs and were therefore treated as unstable, penalty-dependent solutions rather than retained phase boundaries.

**Figure 10:**
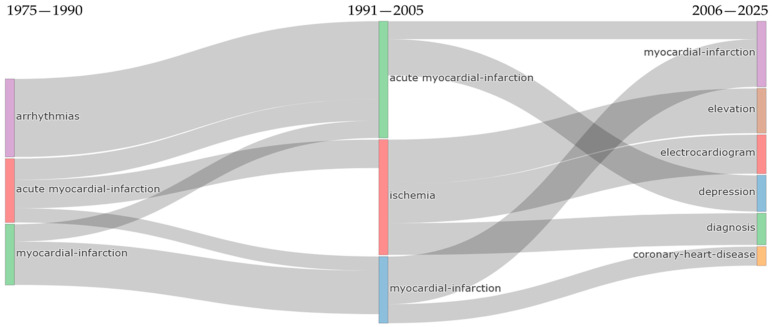
Sankey diagram illustrating the thematic transition from isolated physiological observations (1975–1990) to an integrated biopsychosocial framework (2006–2025).

These breakpoints delineate the 51-year dataset into three distinct developmental phases. Mann–Whitney U tests confirmed significant differences in publication volume across all adjacent phases, with complete rank separation in both comparisons (rank-biserial r = 1.0). Both adjacent comparisons yielded U = 0, indicating complete rank separation, and the magnitude of the transitions was extremely large by standardized effect-size criteria (Hedges’ g = 3.88 for the first transition and 3.95 for the second; Cohen’s d = 3.98 and 4.05, respectively). The 1990 breakpoint marked a significant transition from an early low-output phase to a sustained intermediate growth phase (Median Difference = 169.5; *p* < 0.000001; Cohen’s d = 3.98). The later retained breakpoint marks the strongest transition in publication intensity (Median Difference = 281.5; *p* < 0.000001; Cohen’s d = 4.05), likely reflecting the emergence of the investigated subject. Based on these statistically derived segments, the subsequent thematic evolution analysis (Sankey diagram) is structured into three periods: 1975–1990, 1991–2005, and 2006–2025 (Figure 10).

Between 1975 and 2005, research topics gradually merged from separate physical observations into a single, unified medical approach. Early separate themes like acute myocardial-infarction, arrhythmias, and ischemia combined into one central myocardial-infarction group during the intermediate period (1991–2005). This merge was notable because mental health terms such as ‘anxiety’ and ‘stress’ began appearing alongside cardiac terminology in the same publications, suggesting increasing co-investigation of psychological and cardiovascular dimensions.

In the modern era (2006–2025), the field branched out from a single disease focus into three specialized areas. The largest research trend moved into Coronary-Heart-Disease (227 occurrences), linking heart issues with serious mental health conditions like major depression, PTSD, and panic disorder, along with biological connections like heart-rate-variability. Meanwhile, the Myocardial-Infarction theme shifted to focus on patient well-being, such as quality-of-life and social support, while a separate area focused on treatments like exercise and their efficacy. These thematic shifts in publication keywords are consistent with the bibliometric profile of a maturing research area, with increasing co-occurrence of biological, psychological, and recovery-related terminology.

Research in the 1990s was heavily characterized by a focus on acute physiological mechanisms and immediate interventional strategies. The literature was dominated by terms such as silent myocardial ischemia (Median Year: 1992) and thrombolytic therapy (Median Year: 1999), suggesting a strong publication emphasis on detecting asymptomatic cardiac risks and managing acute clot formation during this period.

**Figure 11:**
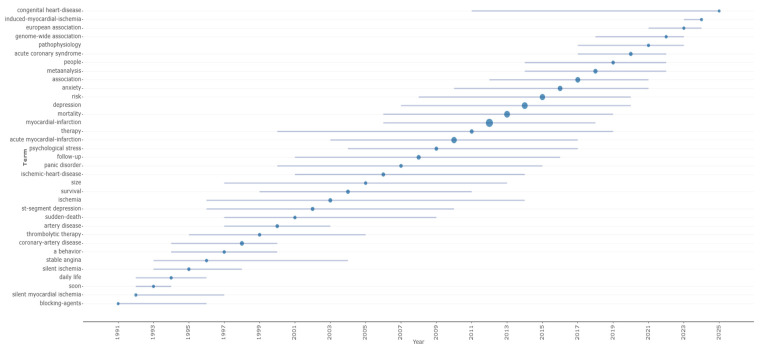
Temporal Evolution of Trend Topics (1991–2025).

As the field moved into the 2000s, the conceptual focus shifted significantly toward diagnostic refinement and long-term epidemiological outcomes (Figure 11). The terminology evolved to emphasize specific diagnostic markers like st-segment depression (Median Year: 2002) and broader chronic disease categories such as ischemic-heart-disease (Median Year: 2006). The emergence of survival (Median Year: 2004) as a central topic indicates a maturing discipline that moved beyond acute event management to prioritize long-term prognosis and survivorship in patients with comorbid cardiovascular and psychiatric conditions.

**Figure 12:**
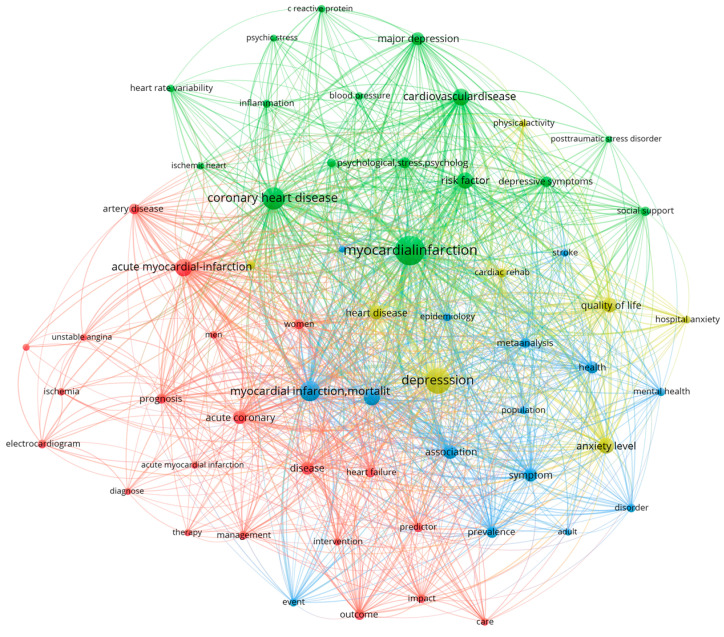
Bibliometric co-occurrence network of high-frequency terms.

The keyword co-occurrence (Figure 12) network visualizes the conceptual structure of the field, revealing a high density of connections between psychiatric and cardiovascular terminologies. The network analysis identifies distinct thematic clusters that represent the core intellectual pillars of the domain. The largest cluster (Group 1) is anchored by foundational cardiovascular terms such as myocardial infarction (occurrences n = 2178), acute myocardial infarction (n = 925), and acute coronary syndrome (n = 463). This cluster also contains significant physiological and prognostic markers including mortality (n = 822), prognosis (n = 460), and atherosclerosis (n = 126), suggesting a primary focus on the clinical mechanisms and fatal outcomes associated with acute coronary events. A second, distinct cluster (Group 2) captures the psychiatric dimension, dominated by depression (n = 2015; encompassing publications using both formal diagnostic criteria and dimensional symptom measures), anxiety (n = 899; similarly spanning diagnosed anxiety disorders and self-reported symptomatology), and stress (n = 323; predominantly reflecting psychological or psychosocial stress rather than a diagnostic entity) indicating a research trend focused on the epidemiological burden and patient-reported outcomes of mental health comorbidities. A third, smaller cluster (Group 3) appears to represent the specific intersection of these domains, featuring terms like depressive symptoms (n = 672) and coronary heart disease (n = 607), further emphasizing the syndromic overlap. The high frequency of the term risk factors (n = 816) across groups suggests it serves as a conceptual bridge, linking the behavioral pathology of the psychiatric cluster with the hard clinical endpoints of the cardiovascular cluster.

## 4. Discussion

This research field has grown from a handful of Western countries to a truly global effort involving 53 nations working in four collaborative networks. The United States dominates in absolute productivity with 5233 documents and the highest total link strength (2480), indicating a well-established research infrastructure with extensive international connectivity. However, the geographic distribution demonstrates that leadership in this interdisciplinary domain extends well beyond volume metrics. The United Kingdom anchors a second major cluster with 1263 documents and robust collaborative strength (1712 TLS), while a Germanic-Nordic cluster cantered on Germany and the Netherlands exhibits particularly strong intra-European research networks. The emergence of an Asia-Pacific cluster, anchored by Japan (422 documents) and China (917 documents), signals the globalization of psychocardiology research, though collaborative integration patterns differ markedly between these contributors.

The four-cluster structure reflects the inherent complexity of investigating mental health–cardiac intersections across diverse healthcare systems, cultural contexts, and population characteristics. Depression manifestations, cardiovascular risk profiles, and treatment responses vary considerably across populations, necessitating research approaches that capture region-specific factors including genetic backgrounds, healthcare delivery models, and cultural attitudes toward mental health. The substantial participation from 53 countries with varying research traditions suggests that the field has matured beyond its origins in Western academic medicine to embrace the global burden of cardiovascular-psychiatric comorbidity. This geographic diversification positions mental health–ACS research to address the worldwide scope of these interconnected conditions, which together represent leading causes of disability and mortality across all income levels.

The striking contrast between publication volume and citation efficiency among leading countries illuminates diverse research strategies within the mental health–ACS intersection. While the United States leads in total citations (345,358), smaller research ecosystems demonstrate disproportionate scientific influence. The Netherlands achieves the highest citation efficiency at 67.54 citations per document despite ranking seventh in volume, followed closely by Canada at 67.08 citations per document. This pattern suggests that strategic positioning within international collaboration networks and focused research programs can amplify scientific impact beyond publication numbers alone. Germany’s balanced profile combining substantial output (753 documents) with strong citation rates (54.06 citations per document) and collaborative strength (1332 TLS), exemplifies how established pharmaceutical and cardiovascular research infrastructure produces consistently influential work.

In contrast, China’s rapid research expansion reveals a volume-impact mismatch characteristic of emerging research ecosystems, with 917 documents generating only 23.34 citations per document and notably limited international collaboration (TLS 194). This pattern, observed across multiple bibliometric domains, likely reflects recent strategic investment in domestic research capacity that has not yet achieved full integration with established international networks. Japan presents a similar profile, with moderate output (422 documents) but exceptionally low collaborative strength (TLS 194), suggesting research programs that operate somewhat independently from global consortia. These geographic patterns indicate that future field advancement will benefit from deliberate efforts to integrate emerging contributors into established collaborative frameworks.

The thematic evolution analysis across three distinct periods (1975–1990, 1991–2005, 2006–2025) reveals a systematic transformation from isolated pathophysiological observations to an integrated biopsychosocial framework for understanding cardiovascular-psychiatric comorbidity. During the foundational period, research remained compartmentalized within discrete domains, acute myocardial infarction, arrhythmias, and basic ischemia research, with limited conceptual integration between cardiac and psychological dimensions. The intermediate period witnessed dramatic thematic expansion as these initially separate streams began converging around shared mechanistic and clinical concerns.

The contemporary period (2006–2025) demonstrates remarkable thematic consolidation, with the largest flow (1084 occurrences) connecting myocardial infarction research across periods through concepts including quality-of-life, social support, cardiac rehabilitation, mental health, anxiety, stress, and antidepressants. This dominant pathway illustrates how post-infarction psychological outcomes have become central to contemporary cardiovascular research rather than peripheral concerns. Equally significant is the substantial flow (604 occurrences) linking acute myocardial infarction with depression research, encompassing survival, management, outcomes, heart failure, and intervention, reflecting an increased scholarly focus on how depression might affect cardiac prognosis.

The emergence of coronary heart disease as a major contemporary theme (227 occurrences) explicitly integrating major depression, heart rate variability, panic disorder, posttraumatic stress disorder, and psychosocial factors signals the field’s maturation toward recognizing psychiatric conditions as mechanistically linked comorbidities rather than merely psychological reactions to cardiac events. The inclusion of serotonin reuptake inhibitors within this thematic cluster further indicates that pharmacological management of depression in cardiac patients has become a mainstream research concern. This thematic convergence mirrors a shift in the literature, wherein cardiovascular disease is increasingly discussed as a systemic condition, with growing publication emphasis on the potential bidirectional interactions between psychological, inflammatory, and behavioural factors.

The PELT changepoint detection analysis identified two statistically robust breakpoints at 1990 and 2005, demonstrating 100% consistency across sensitivity analyses and confirming distinct developmental phases in mental health–ACS research. The 1990 breakpoint should be interpreted cautiously. Although this transition coincides with the emergence of influential epidemiological studies that helped establish depression as an independent cardiovascular risk factor, our metadata-completeness sensitivity analysis indicates that the discontinuity also reflects historical changes in WoSCC indexing practices. In particular, author keywords and Keywords Plus were extremely sparse in pre-1991 records and increased sharply thereafter, which would have enhanced Topic-field retrievability independently of genuine growth in the literature. Accordingly, the 1990–1991 inflection is best understood as a combined bibliographic and intellectual transition rather than as a purely substantive surge in research activity. The 2005 breakpoint represents the most critical transition (Cohen’s d = 4.05, *p* < 0.000001), marking the onset of exponential growth that would see annual output increase from 349 publications to over 600 by 2022.

The intellectual foundation of this field was established through seminal prognostic investigations that transformed depression from an incidental comorbidity into a recognized cardiovascular risk factor. Frasure-Smith and colleagues’ pioneering 1993 study demonstrated that major depression following myocardial infarction conferred a hazard ratio of 5.74 for 6-month cardiac mortality, an effect magnitude comparable to left ventricular dysfunction [30]. Their subsequent 18-month follow-up confirmed persistent risk elevation (adjusted OR 6.64, 95% CI 1.76–25.09), with mortality concentrated among depressed patients exhibiting ≥10 premature ventricular contractions per hour, suggesting arrhythmic mechanisms [31]. These foundational observations catalyzed meta-analytic consolidation: Barth et al. synthesized 20 studies demonstrating that depressed coronary heart disease patients faced two-fold mortality risk within two years (OR 2.24, 95% CI 1.37–3.60), with effects persisting after adjustment for traditional risk factors (adjusted HR 1.76, 95% CI 1.27–2.43) [32]. The INTERHEART study extended these findings globally, documenting psychosocial factors (stress, depression, locus of control) across 11,119 cases and 13,648 controls from 52 countries, with permanent work stress conferring OR 2.14 (99% CI 1.73–2.64) and collective psychosocial factors accounting for 32.5% population-attributable risk [33].

Landmark intervention trials such as ENRICHD, SADHART, COPES, and CODIACS remain important because they marked the transition from observational associations to implementable care models in post-ACS/post-MI populations. However, within a bibliometric discussion their main value is historical and thematic: they show how the field moved from proving prognostic relevance toward testing collaborative care, pharmacological safety, and the limits of universal screening. Collectively, these studies demonstrated that depressive symptoms can be addressed safely after ACS, but that symptom improvement does not consistently translate into durable cardiovascular benefit, thereby helping explain why later literature shifted toward risk stratification, phenotyping, and targeted follow-up rather than universal screening alone [34,35,36,37,38,39].

Recent literature broadens this picture beyond inflammatory phenotyping [40,41]. Earlier syntheses had already suggested high depression prevalence and elevated suicidality after ACS [42], but newer statements and meta-analyses place greater emphasis on the broader burden of post-ACS/post-MI psychological distress and on the need for structured longitudinal assessment [43,44]. Consistently, a contemporary ACS cohort found that clinician impression alone had low sensitivity (32%) relative to PHQ-9 screening, while 20.7% of patients screened above the cutoff for clinical depression, supporting the use of validated screening tools rather than unaided clinical judgment [45]. Consistently, a contemporary ACS cohort found that clinician impression alone had low sensitivity (32%) for detecting depression compared with PHQ-9-based assessment, despite 20.7% of patients screening above the clinical threshold, supporting the use of validated screening tools rather than unaided clinical judgment [9]. This aligns with the 2025 ESC Clinical Consensus Statement on mental health and cardiovascular disease, which recommends screening after a new cardiovascular event, during follow-up, and whenever clinically indicated, with brief two-item instruments followed by longer validated tools when positive [46]. Moreover, a 2025 cohort study found that in ACS patients, life stressors in the presence of suicidal ideation at two weeks were associated with poorer long-term major adverse cardiac outcomes, supporting early psychosocial assessment after ACS [47]. Taken together, these newer studies suggest that the contemporary research frontier is less about re-establishing whether depression matters and more about identifying high-risk subgroups, improving detection, and integrating mental health surveillance into post-ACS care pathways.

### 4.1. Limitations

This bibliometric investigation provides insight into the evolution of mental health–ACS research, but several methodological limitations should be acknowledged. The reliance on WoSCC as the single data source represents a methodological trade-off between comprehensive coverage and analytical precision. Because the analysis was limited to WoSCC, relevant studies not indexed in this database, particularly regional, non-core, or less visible literature, may be underrepresented. Therefore, the findings should be interpreted as a mapping of the WoSCC-indexed literature rather than the full body of scholarship on the topic. Beyond this single-source constraint, WoSCC exhibits well-documented structural biases in journal selection that disproportionately favor English-language periodicals and journals published in countries hosting major academic publishers (e.g., the United States, the United Kingdom, and the Netherlands), while journals from other countries and languages remain underrepresented [17]. Furthermore, the geographical representation of a field in the WoSCC can be skewed because its indexing depth varies significantly by region, country, and language. Historical gaps in author metadata may also affect longitudinal analyses of institutional and international collaboration, as older WoS records may contain missing address information [48].

An additional limitation concerns long-range Topic-based retrieval in WoSCC, because older records show historically uneven availability of non-title metadata, particularly author keywords and Keywords Plus. Therefore, early publication trends, especially around 1990–1991, may partly reflect changes in metadata coverage rather than only changes in research activity. As this is a bibliometric study, the identified breakpoints, citation patterns, and keyword co-occurrence structures should be interpreted as indicators of publication activity and thematic emphasis within the indexed literature, rather than as direct evidence of changes in clinical practice, guideline adoption, or causal mechanisms. References to landmark clinical trials are included to contextualize the literature historically. However, this analysis cannot determine whether those trials caused subsequent shifts in publication trends. As with bibliometric indicators more broadly, which measure citation impact rather than real-world clinical significance, bibliometric mapping cannot evaluate the diagnostic validity of the mental health constructs represented in the indexed literature or determine the treatment efficacy of the interventions discussed across publications [49]. Similarly, the keyword and thematic analyses reflect the terminology used by source authors rather than independently verified diagnostic classifications. The co-occurrence of a term such as “depression” in the keyword network does not distinguish between studies that applied structured clinical interviews conforming to DSM-5 criteria and those that relied on self-report symptom inventories or used the term descriptively. This granularity of diagnostic operationalization falls outside the scope of bibliometric mapping and would require a complementary systematic review with individual-study quality assessment to characterize fully.

Regarding the changepoint detection framework, several statistical caveats apply. The Mann–Whitney U tests used to validate the PELT-derived segments assume independence of observations within each segment. Annual publication counts in a growing field are inherently autocorrelated; high output in one year predicts high output in the next, which may inflate the nominal significance of the reported *p*-values. However, the complete rank separation between all adjacent segments (rank-biserial r = 1.0) and the very large effect sizes (Cohen’s d > 3.9) indicate that the detected transitions are substantive and not merely artifacts of statistical dependence. The segment-level sample sizes (n = 15–20 annual observations per phase) are modest, introducing uncertainty into the effect size estimates; accordingly, Cohen’s d values should be interpreted as indicating the approximate order of magnitude of each transition rather than as precise point estimates. Finally, the analysis relied on a single changepoint detection algorithm (PELT with RBF kernel); although the breakpoints proved robust across the tested penalty range, confirmation with alternative segmentation approaches, such as Bayesian changepoint detection, could further strengthen confidence in the identified temporal boundaries.

Interpretation of citation metrics for publications from 2020 to 2025 requires caution, as these records have had less time to accumulate citations. This citation lag may undervalue emerging research directions, including digital health interventions, precision psychiatry, and post-pandemic developments at the interface of cardiovascular and mental health research. More generally, bibliometric analysis captures publication patterns rather than study quality, clinical implementation, or patient outcomes.

### 4.2. Future Research Directions

The present bibliometric mapping indicates several priorities for future research at the psychiatry–cardiology interface. First, more clinically granular work is needed to distinguish formal psychiatric diagnoses from symptom-level constructs that are frequently grouped together in the indexed literature under broad terms such as depression, anxiety, distress, or mental health. Future primary studies should therefore use clearer phenotypic definitions, repeated psychiatric assessments across the acute and recovery phases after ACS, and standardized reporting frameworks that permit stronger comparison across cohorts.

Second, the thematic shift identified in recent years suggests that the next phase of the field will likely depend on better integration of psychopathology with mechanistic and prognostic stratification. In particular, future studies should examine how depressive, anxiety-related, trauma-related, and stress-related phenotypes interact with inflammatory signaling, autonomic dysfunction, platelet reactivity, sleep disruption, and behavioral adherence pathways in order to identify clinically meaningful high-risk subgroups. This would help move the field beyond broad associations toward more precise risk models and more targeted intervention strategies.

Third, intervention research should continue to move from universal screening paradigms toward enriched or stratified models of care. Collaborative-care pathways, stepped psychiatric follow-up, digital monitoring, and hybrid cardiology–mental health models may be especially relevant directions, but their value should be tested in subgroups defined by symptom persistence, suicidality, inflammatory burden, or impaired functional recovery rather than in undifferentiated post-ACS populations alone. Greater attention should also be given to underrepresented regions and healthcare systems, because current bibliometric patterns remain dominated by high-income countries and English-language indexing.

From a methodological perspective, future bibliometric work could strengthen this literature by comparing WoSCC-derived trends with parallel analyses from other databases, by validating temporal segmentation with complementary changepoint approaches, and by expanding reproducibility practices through the sharing of scripts, keyword-normalization resources, and derived non-licensed outputs whenever legally feasible.

## 5. Conclusions and Prospects

This bibliometric analysis documents major changes in publication activity and thematic structure at the intersection of mental health and ACS. Over five decades, the indexed literature has expanded from sparse early publications to a much larger and more thematically differentiated body of research. The application of PELT changepoint detection identified two critical inflection points 1990 and 2005 that demarcate specific developmental phases. The 1990 breakpoint signaled an important transition in the field, but this inflection should be interpreted as reflecting both genuine conceptual development and improved Topic-field metadata availability in WoSCC around 1990–1991. The 2005 breakpoint marked the onset of exponential growth, where annual output surged from 349 to over 600 publications by 2022, consistent with increasing publication activity and scholarly attention within cardiovascular journals.

Historically, landmark investigations helped shape the literature by framing depression as a potentially important cardiovascular risk marker rather than only an incidental comorbidity. Seminal prognostic studies reported hazard ratios for cardiac mortality between 2.24 and 6.64 in post-myocardial infarction populations, with effects persisting after adjustment for conventional risks. While global validation established population-attributable risks exceeding 30%, the translation of this evidence into practice proved complex. Major randomized controlled trials failed to demonstrate that treating depression automatically improved cardiac outcomes, and this period was followed by increased publication attention to mechanistic pathways such as heart rate variability, inflammation, and autonomic dysfunction linking psychiatric status to cardiovascular pathophysiology.

Thematic analysis indicates a shift in research emphasis, from publications focused primarily on psychological reactions to cardiac events toward publications that investigate depression, anxiety, and PTSD as potential mechanistic contributors to cardiovascular outcomes. This terminological progression may also reflect changing indexing and publication language over time, in addition to substantive shifts in research emphasis. Contemporary themes now prioritize quality of life, cardiac rehabilitation, and biopsychosocial care models. Looking forward, the field is poised to leverage these foundations for precision psychiatry, focusing on subgroup identification and the implementation of evidence-based interventions. despite limitations such as single-database constraints, the documented growth trajectory suggests that this domain will continue to fundamentally reshape cardiovascular care delivery in an era increasingly defined by the inseparability of mental and physical health. Furthermore, while increasing keyword co-occurrence suggests growing scholarly interest in specific mechanistic links (such as inflammation, autonomic dysfunction, or platelet reactivity), these bibliometric patterns reflect publication trends and cannot substitute for clinical validation of these biological pathways.

## Figures and Tables

**Table 1:** Global research leadership in mental health–acute coronary syndrome research (national productivity, scientific influence, and collaborative engagement).

Country	Documents	Citations	Average Citations/Document	TLS
USA	5233	345,358	66.00	2480
United Kingdom	1263	81,925	64.87	1404
Canada	955	64,062	67.08	1064
China	917	21,404	23.34	532
Germany	741	45,259	61.08	839
Netherlands	736	49,711	67.54	799
Australia	686	31,820	46.38	747
Sweden	609	29,600	48.60	729
Italy	595	29,651	49.83	644
Japan	422	12,547	29.73	194

TLS, total link strength.

**Table 2:** Citation metrics, productivity indices, and publication history of leading journals in mental health–ACS research.

Source	h-Index	g-Index	m-Index	Total Citations	Publications	Publication Start
Circulation	101	194	2.02	37,818	210	1977
Psychosomatic Medicine	99	178	2.415	36,408	353	1986
Journal of the American College of Cardiology	74	140	1.682	20,792	195	1983
American Journal of Cardiology	71	113	1.543	17,837	358	1981
Journal of Psychosomatic Research	64	102	1.255	13,738	279	1976
American Heart Journal	60	91	1.176	11,383	254	1976
Archives of Internal Medicine	56	71	1.12	12,076	71	1977
European Heart Journal	54	96	1.256	10,341	170	1984
Jama-Journal of the American Medical Association	42	45	1.00	16,734	45	1985
International Journal of Cardiology	41	60	0.976	5144	198	1985

h-index, Hirsch index; g-index, Egghe’s g-index; m-index, m-quotient.

**Table 3:** Ranking of the top 10 documents by total global citations and normalized citation scores.

Paper/Source	TC	TC/Year	Normalized TC	DOI
HOLT-LUNSTAD J, 2010, PLOS MED	4926	289.76	63.00	https://doi.org/10.1371/journal.pmed.1000316
LINK BG, 1995, J HEALTH SOC BEHAV	4272	133.50	48.68	https://doi.org/10.2307/2626958
LESCH KP, 1996, SCIENCE	4114	132.71	34.70	https://doi.org/10.1126/science.274.5292.1527
SIMOPOULOS AP, 2002, BIOMED PHARMACOTHER	2985	119.40	24.78	https://doi.org/10.1016/S0753-3322(02)00253-6
HAWKLEY LC, 2010, ANN BEHAV MED	2692	158.35	34.43	https://doi.org/10.1007/s12160-010-9210-8
O’DONNELL MJ, 2010, LANCET	2421	142.41	30.96	https://doi.org/10.1016/S0140-6736(10)60834-3
RAISON CL, 2006, TRENDS IMMUNOL	2360	112.38	33.26	https://doi.org/10.1016/j.it.2005.11.006
CORRIGAN P, 2004, AM PSYCHOL	2291	99.61	25.50	https://doi.org/10.1037/0003-066X.59.7.614
HOWREN MB, 2009, PSYCHOSOM MED	2290	127.22	36.75	https://doi.org/10.1097/PSY.0b013e3181907c1b
MOSS-MORRIS R, 2002, PSYCHOL HEALTH	2267	90.68	18.82	https://doi.org/10.1080/08870440290001494

TC, total citations.

## Data Availability

The original contributions presented in this study are included in the article. Further inquiries can be directed to the corresponding author.

## Abbreviations

The following abbreviations are used in this manuscript:

ACS	Acute coronary syndromes
WoS	Web of Science Core Collection
PTSD	Post-traumatic stress disorder
OCD	Obsessive–compulsive disorder
NSTEMI	Non-ST-segment elevation myocardial infarction
STEMI	ST-segment elevation myocardial infarction
AMI	Acute myocardial infarction
TS	Topic (Web of Science field)
PELT	Pruned Exact Linear Time
UMLS	Unified Medical Language System
MeanTCperArt	Mean Total Citations per Article
TLS	Total link strength
RBF	Radial Basis Function
TC	Total citations
MI	Myocardial infarction
HRSD	Hamilton Rating Scale for Depression
SSRI	Selective serotonin reuptake inhibitor
CGI-I	Clinical Global Impression–Improvement
OR	Odds ratio
HR	Hazard ratio
CI	Confidence interval
